# Population Genetic Structure of the Tropical Two-Wing Flyingfish (*Exocoetus volitans*)

**DOI:** 10.1371/journal.pone.0163198

**Published:** 2016-10-13

**Authors:** Eric A. Lewallen, Andrew J. Bohonak, Carolina A. Bonin, Andre J. van Wijnen, Robert L. Pitman, Nathan R. Lovejoy

**Affiliations:** 1 Department of Biological Sciences, University of Toronto Scarborough, Toronto, Ontario, Canada; 2 Departments of Biochemistry & Molecular Biology and Orthopedic Surgery, Mayo Clinic, Rochester, Minnesota, United States of America; 3 Department of Biology, San Diego State University, San Diego, California, United States of America; 4 University of St. Thomas, St. Paul, Minnesota, United States of America; 5 Southwest Fisheries Science Center, National Marine Fisheries Service, National Oceanic and Atmospheric Administration, La Jolla, California, United States of America; University of California Santa Cruz, UNITED STATES

## Abstract

Delineating populations of pantropical marine fish is a difficult process, due to widespread geographic ranges and complex life history traits in most species. *Exocoetus volitans*, a species of two-winged flyingfish, is a good model for understanding large-scale patterns of epipelagic fish population structure because it has a circumtropical geographic range and completes its entire life cycle in the epipelagic zone. Buoyant pelagic eggs should dictate high local dispersal capacity in this species, although a brief larval phase, small body size, and short lifespan may limit the dispersal of individuals over large spatial scales. Based on these biological features, we hypothesized that *E*. *volitans* would exhibit statistically and biologically significant population structure defined by recognized oceanographic barriers. We tested this hypothesis by analyzing cytochrome b mtDNA sequence data (1106 bps) from specimens collected in the Pacific, Atlantic and Indian oceans (n = 266). AMOVA, Bayesian, and coalescent analytical approaches were used to assess and interpret population-level genetic variability. A parsimony-based haplotype network did not reveal population subdivision among ocean basins, but AMOVA revealed limited, statistically significant population structure between the Pacific and Atlantic Oceans (Φ_ST_ = 0.035, p<0.001). A spatially-unbiased Bayesian approach identified two circumtropical population clusters north and south of the Equator (Φ_ST_ = 0.026, p<0.001), a previously unknown dispersal barrier for an epipelagic fish. Bayesian demographic modeling suggested the effective population size of this species increased by at least an order of magnitude ~150,000 years ago, to more than 1 billion individuals currently. Thus, high levels of genetic similarity observed in *E*. *volitans* can be explained by high rates of gene flow, a dramatic and recent population expansion, as well as extensive and consistent dispersal throughout the geographic range of the species.

## Introduction

Understanding the genetic population structure of marine fishes is critical not only for documenting genetic diversity, speciation, and evolution, but also to inform global fisheries management efforts. Recent and rapid losses of global marine biodiversity make the genetic characterizations of marine organisms, and populations, a high priority [1]. In general, coastal and reef associated fish species are best studied [2, 3], probably because of their proximity to human populations and corresponding ease of sampling. Less is known about the population-level genetic diversity of organisms inhabiting the epipelagic zone (pelagic surface waters), one of the largest habitats on Earth (>130 million km^2^; [4]). This is in part due to the difficulty of sampling such vast geographic areas, and frequent challenges with species delimitation [5].

Because epipelagic habitats (surface waters of the open ocean) are among the most vulnerable to increasing sea surface temperatures and global climate change, it is imperative that we understand the population-level genetic diversity of open ocean inhabitants. Spatial patterns of genetic variation have been reviewed for populations of epipelagic and circumtropical fishes [5]. A number of traits are shared by many circumtropical species, including broadcast spawning, brevity of larval duration, and large adult range. Key questions that remain to be addressed are how circumtropical species achieve global geographic ranges and maintain global population connectivity. Population genetic investigation of the Tropical Two-Wing Flyingfish, *Exocoetus volitans*, (family Exocoetidae), may be particularly useful for resolving these questions.

Like other members of the Exocoetidae, *E*. *volitans* can leap from the water and glide through the air using enlarged pectoral fins, allowing evasion of epipelagic predators. Despite an exceptional predator-evasion strategy, flyingfishes are important prey for larger fish, sharks, squids, seabirds and marine mammals. As an epipelagic specialist, *E*. *volitans* completes all life stages in the upper stratum of the water column, and can be abundant in warm surface waters [6]. The patchy abundance of *E*. *volitans* at smaller spatial scales is likely due to extrinsic factors, such as primary productivity [7].

*Exocoetus volitans* spawns buoyant, pelagic eggs that should contribute to increased dispersal potential [8] and high gene flow. However, other life history traits such as short larval phase, coupled with a brief hatching time of approximately 1–2 weeks [9], and one-year life span [7] should limit population connectivity, especially on global scales. Further, *E*. *volitans* is small bodied (<210 mm; [6]), a relatively slow swimmer, and incapable of swimming great distances [10]. These characteristics suggest that *E*. *volitans* could be subdivided into discrete populations throughout its geographic range.

In this study, we tested for population subdivision using mtDNA sequences of *E*. *volitans* from throughout its entire circumtropical range, including far offshore epipelagic localities that are only accessible for research via dedicated long-range expeditions. Specifically, we addressed the following questions: (i) What is the worldwide genetic population structure of *E*. *volitans*? (ii) Are populations of *E*. *volitans* separated by previously described marine fish barriers? (iii) Is there evidence of a recent population expansion, or has the effective population size remained constant for a long period (i.e., drift-mutation equilibrium)? Using parsimony, AMOVA, Bayesian, and coalescent modelling methods, we measured and interpreted multiple population genetic parameters for this representative circumtropical epipelagic specialist

## Materials and Methods

### Specimen collection

**Fig 1:**
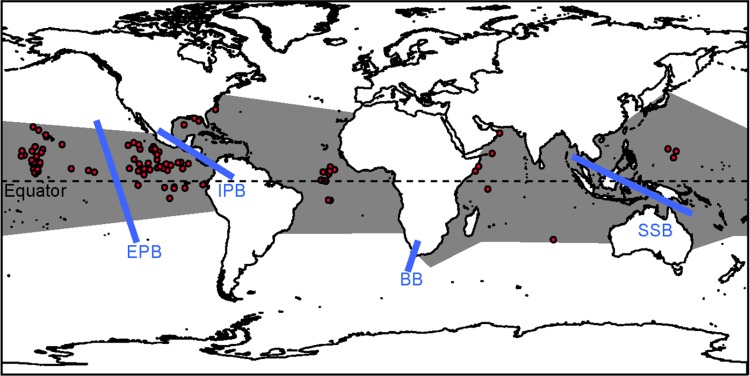
Collection localities for *Exocoetus volitans* specimens used in this study. In total, 266 individuals were collected from 97 localities **(red circles)**. Grey polygons approximate the species' distribution, based on Parin and Shakhovskoy [6]. Previously-described marine barriers tested are indicated by blue bars (EPB = Eastern Pacific Barrier; IPB = Isthmus of Panama Barrier; BB = Benguela Barrier; SSB = Sunda Shelf Barrier). This figure was generated using ArcGIS, version 9.3.1 (ESRI, Redlands, CA, USA).

*Exocoetus volitans* specimens were collected at night using flood lights and long-handled dip nets during 13 research cruises on the following research vessels from 1992 to 2010: *Endeavor*, *Shoyo Maru*, *Gordon Gunter*, *McArthur II*, *David Starr Jordan*, *Kahana*, and *Oscar Elton Sette*. The majority of specimens in the Pacific Ocean were collected during *Stenella Abundance Research* cruises, while Atlantic specimens were collected during the *South Atlantic Black Carbon* research cruise. Specimens were also donated by collaborators. In total, 266 *E*. *volitans* specimens were collected from 97 locations throughout the Atlantic, Pacific, and Indian Oceans (Table 1, Fig 1, S1 Table). All specimen collections were performed in accordance with all ethical care and animal welfare standards. No specific permissions or field permits were required for any of the localities where specimens were collected because these areas and species are not protected. In particular, these field studies did not involve endangered or protected species. All specimens were euthanized using a seawater ice bath to minimize undue pain and stress to the animals. Tissues for DNA analysis were removed post-mortem and preserved in 95% ethanol. Also post-mortem, whole-specimen vouchers were frozen in seawater, fixed in 10% formalin, and cataloged at the Royal Ontario Museum, Scripps Institution of Oceanography, and Los Angeles County Museum. This study was not conducted on private land, in a national park, or protected area of the land or sea.

**Table 1:** Summary statistics from population genetic analyses of *Exocoetus volitans* divided by ocean basin, and population clusters (northern and southern), and overall. S = number of variable sites; h = haplotype diversity; π = nucleotide diversity.

Locality	n	S	# of mutations	Unique Haplotypes	h (SD)	G + C Content	π (SD)	Avg. # of nucleotide differences	θ_k_ (per sequence)	θ_s_ (per site)
**Worldwide**	266	258	281	242	0.998 (0.001)	0.424	0.00637 (0.00020)	7.049	44.626	0.04125
**Pacific Ocean**	111	170	179	105	0.999 (0.002)	0.424	0.00731 (0.00029)	8.088	33.887	0.03064
**Atlantic Ocean**	150	187	198	135	0.998 (0.001)	0.424	0.00552 (0.00025)	6.104	35.455	0.03206
**Indian Ocean**	5	15	15	4	0.900 (0.161)	0.426	0.00561 (0.00151)	6.200	7.200	0.00651
**Northern cluster**	183	216	228	167	0.998 (0.001)	0.424	0.00584 (0.00023)	6.456	39.419	0.03564
**Southern cluster**	83	141	144	80	0.999 (0.002)	0.424	0.00733 (0.00035)	8.104	28.858	0.02609

### Genetic data collection

Genomic DNA was extracted using DNeasy kits (Qiagen, Hilden, Germany) and a portion of the mitochondrial cytochrome *b* (cytb) gene (1106 bps) was amplified using previously published protocols [11]. Cytb is a commonly used molecular marker of genetic diversity, facilitating comparison with other studies. Cytb has also been used previously to resolve the phylogenetic relationships among flyingfish species [11]. Sequences were edited and aligned using Sequencher v.4.6 (Gene Codes Corporation, Ann Arbor, MI), and have been deposited in Genbank (Accession numbers HQ325634, HQ325635, KX912952, KX913215).

### Summary statistics and haplotype network

Population genetic summary statistics (number of haplotypes; haplotype diversity, *h*; nucleotide diversity, *π*; Θ_k_ (per sequence) and Θ_s_ (per site) were calculated using DNAsp v.5 [12]. To determine the relationships of mtDNA haplotypes, a cytb gene genealogy was generated using the statistical parsimony method implemented by TCS v1.21 [13]. We resolved ambiguous mutational relationships in the network using the criteria of Crandall, Templeton & Sing [14] and Templeton, Routman & Phillips [15].

### Analyses of molecular variance

Because biogeographic barriers for epipelagic fishes are not well characterized, we postulated that barriers which have been demonstrated in other broadly distributed marine fishes could affect *E*. *volitans*. Globally, seven major biogeographic features limit gene flow among marine fishes, and have driven allopatric speciation events in numerous marine taxa [3]. Four of these barriers were relevant to our sampling scheme and the geographic range of *E*. *volitans*: the Isthmus of Panama Barrier (physically separates the Atlantic and Pacific Oceans, Central America), Sunda Shelf Barrier (numerous islands that create a division between the Indian and Pacific Oceans, southeast Asia), Benguela Barrier (a region of converging water currents between the Atlantic and Indian Oceans, South Africa), and the Eastern Pacific Barrier: a deep water division between the Western and Eastern Pacific (Fig 1). The Eastern Pacific and Benguela barriers are oceanic, can quickly change size and location depending on the year or season, and are therefore expected to be permeable for *E*. *volitans*. This is not the case for terrestrial barriers, such as the Isthmus of Panama, and the semi-terrestrial Sunda Shelf barrier. Despite the dynamic nature of oceanic barriers, we opted to include an assessment of all four potential barriers to flyingfish dispersal. To quantify genetic divergence among populations, defined *a priori* by collection locality, we conducted Analysis of Molecular Variance (AMOVA) within Arlequin v.3.5 [16] separately for each of the four barriers listed above (AMOVA analysis parameters: deletions, transitions, and transversions = 1; allowed missing data = 0.05; molecular distance = pairwise difference; distance matrix = minimum spanning network (inter-haplotypic); permutations = 10,000). All samples adjacent to these barriers were used in AMOVA comparisons.

### Bayesian clustering analysis

MOVA requires that individuals are grouped *a priori* into testable gene pools. To delineate populations more objectively, we used the Bayesian clustering model implemented by Geneland v.1 [17, 18]. To reduce the potential impact caused by multiple individuals collected at the same locality, we set the spatial uncertainty of collection sites to 0.1 Universal Transverse Mercator. Ten million MCMC generations were run, with a thinning of 1,000, and the first 5,000 (50%) saved trees were discarded as burn-in. The conservative method for agglomerative "bottom up" clustering was specified by setting the maximum allowable gene pool number to match the total number of samples collected (n = 266). AMOVA was used to quantify genetic differentiation among the clusters identified by Geneland, (using the methods described above). Additionally, to gain a better understanding of the relative position of an equatorial barrier and balance sample sizes, we divided individuals into collection localities north and south of three latitudinal lines (0°N, 5°N, and 7°N) and conducted AMOVAs independently on each data partition.

### Historic population growth

The cytb genealogy was evaluated against the null model of neutral evolution in a single gene pool using three methods described below. Individuals were grouped in putative populations according to distribution (worldwide, Pacific Ocean, Atlantic Ocean, Indian Ocean), as well as based on Bayesian cluster analysis ("northern cluster", and "southern cluster", see Results). First, DNAsp v.5 [12] was used to calculate three metrics commonly referred to as “neutrality tests”: Tajima's D [19] (which compares average divergence to the number of segregating sites), Fu and Li's D [20] (which compares the number of singleton mutations to the number of segregating sites), and Fu and Li's F [20] (which compares the number of unique haplotypes, the number of singleton mutations, and the number of segregating sites). Rejection of the null model in these tests may be due to natural selection, or a violation of the model assumptions (e.g., stable population size throughout the coalescent, population subdivision). Second, genealogy shape was analyzed using a mismatch distribution (MMD), which summarizes the number of mismatches between all possible pairs of sequences in a histogram [21]. We evaluated the demographic MMD using a test in Arlequin v.3.5 [16] for which the null hypothesis is range expansion during the time represented by the gene genealogy. Statistical significance was assessed using 1,000 bootstraps. We assumed the mutation rate (*μ*) was 2% per base pair (bp) per million years, which has been previously used for marine teleosts [22–24]. *Exocoetus volitans* lives for approximately one year [7], which was used as the estimate of generation time. The timing of population expansion was estimated as Tau (*τ*), which was converted to years based on *τ* = 2*μkt* (*k* = number of nucleotides assayed, 1106 bp; *μ* = mutation rate per nucleotide, 2 x 10^−8^; *t* = time since population expansion). Goodness-of-fit between the observed and null MMD was calculated using Harpending raggedness indices (HRI) and sum of square deviations (SSD). Effective population sizes before and after population expansion were estimated as Θ_k_, which was converted to *N*_*e*_ based on Θ_k_ = 2*N*_*e*_*kμ*. Finally, we used BEAST v.1.6.1 [25] to generate a Bayesian skyline plot (BSP), which estimates *N*_*e*_ through time from the shape of the gene genealogy [26] (10 million MCMC generations, GTR + I + Γ model of evolution, random starting tree, 1 tree saved every 1,000 generations, 10% discarded as burn-in). TRACER v.1.4 [27] was used to visualize the BSP.

## Results

### Sampling and Genetic Data

We sequenced cytb fragments (1106 bps) from 266 *E*. *volitans* specimens collected from 97 locations: 150 individuals from 22 locations in the Atlantic Ocean, 111 individuals from 70 locations in the Pacific Ocean, and 5 individuals from 5 locations in the Indian Ocean (Table 1; Fig 1; S1 Table). There were 258 polymorphic sites, and 281 total mutations. Overall haplotype diversity was high (*h* = 0.998 ± 0.001), with 242 singleton haplotypes and only 12 haplotypes shared among individuals. However, nucleotide diversity was low, with an average number of only 7.049 differences among pairs of sequences (*π* = 0.006 ± 0.0002). Global estimates of Θ_k_, and Θ_s_ were 44.626 and 0.04125, respectively (Table 1). Independent analyses of specimens collected within the Pacific, Atlantic and Indian Oceans resulted in similar estimates of haplotype diversity (0.900–0.999). G/C content was 0.424 (and 0.424–0.426 within the three oceans). Genetic diversity metrics that are known to be biased by small sample size tended to be lower in the Indian Ocean, where only 5 individuals were collected (Table 1).

### Gene genealogy

**Fig 2:**
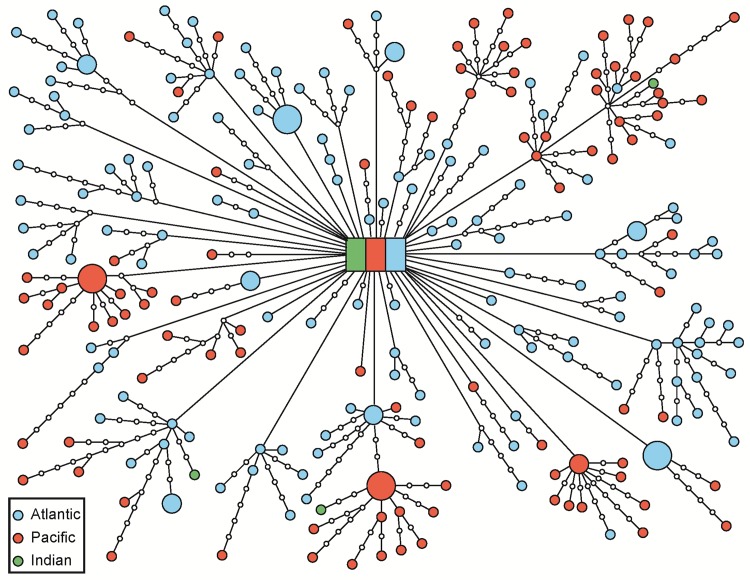
Cytochrome b gene haplotype network. This figure was obtained using statistical parsimony analysis within TCS, v.1.21 [13]. Circle sizes are proportional to the number of shared haplotypes. Lines connecting circles represent single mutations. Blue circles = Atlantic Ocean, Red circles = Pacific Ocean, and Green circles = Indian Ocean. The square in the center represents the ancestral haplotype shared by samples from all three oceans. Open circles represent un-sampled haplotypes.

The gene genealogy revealed a well-connected set of 242 unique, 12 shared, and 324 unsampled haplotypes (Fig 2). The ancestral haplotype (inferred based on its high frequency and centrality [13]) was shared by 10 individuals that were sampled in all three oceans. All other shared haplotypes were restricted to single oceans (8 within Atlantic, 3 within Pacific; Fig 2). The gene genealogy shows that many lineages are composed of individuals from multiple oceans; supporting the idea that gene flow occurs between oceans. Three haplotypes (6255, 6299, and 8387) could not be unambiguously connected and are not shown in Fig 2. An analysis using DNAPARS within PHYLIP v3.69 [28] produced a very similar topology (not presented here).

### Analyses of molecular variance

We found low but statistically significant differentiation across the Isthmus of Panama (Φ_ST_ = 0.0352, p < 0.001), but not the other three putative barriers (Sunda Shelf, Benguela, and Eastern Pacific; Φ_ST_ < 0.0001 and p > 0.4 for each; Table 2). We also detected a significant effect of a worldwide equatorial barrier (see below). The limited number of samples from the Indian Ocean (n = 5) did not allow for robust analysis of the potential influence of the Sunda Shelf and Benguela barriers. Our analyses of the other barriers were more robust, because of higher numbers of samples (n > 45) for each putative population.

**Table 2:** Results of analysis of molecular variance (AMOVA) for cytochrome b sequence data between putative populations of *Exocoetus volitans*. Collection localities, numbers of individuals (n), putative dispersal barriers, Φ_ST_ values, and p-values are listed. Significant results are highlighted in bold and any negative Φ_ST_ values were set to zero.

Putative Population 1	Putative Population 2	Putative Dispersal Barrier	Ф_ST_	p-value
Pacific (n = 111)	Indian (n = 5)	Sunda Shelf Barrier	0.0000	0.944
Atlantic (n = 150)	Indian (n = 5)	Beguela Barrier	0.0000	0.486
Atlantic (n = 150)	Pacific (n = 111)	Isthmus of Panama Barrier	**0.0352**	**0.000**
Eastern Pacific; east of -130.0° (n = 48)	Western Pacific; west of -130.0° (n = 63)	Eastern Pacific Barrier	0.0000	0.709
Northern cluster (n = 183)	Southern cluster (n = 83)	Equatorial Barrier	**0.0265**	**0.000**
North of Equator; 0° worldwide (n = 249)	South of Equator; 0° worldwide (n = 17)	Equatorial Barrier	0.0000	0.568
North of 5°N worldwide (n = 138)	South of 5°N worldwide (n = 128)	Equatorial Barrier	**0.0121**	**0.000**
North of 7°N worldwide (n = 76)	South of 7°N worldwide (n = 190)	Equatorial Barrier	**0.0188**	**0.000**

### Bayesian clustering analysis

**Fig 3:**
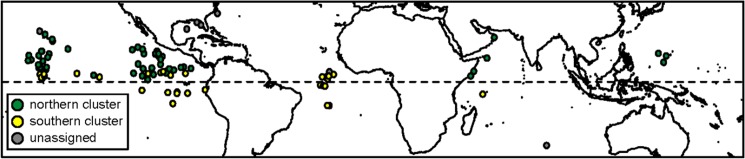
Map of *Exocoetus volitans* population assignments. After cluster analysis using Geneland v.1 [17], posterior probabilities of assignment were loaded into ArcGIS, version 9.3.1 (ESRI, Redlands, CA, USA). Green circles = individuals assigned to the "Northern cluster", Yellow circles = individuals assigned to "Southern cluster", and Grey circles = individuals with low confidence in cluster assignment (posterior probability values < 0.525).

The individual clustering algorithm implemented in Geneland (which does not use *a priori* groups) revealed two gene pools: a “northern cluster” (n = 83: average posterior probability of assignment = 0.855 ± 0.136), and a “southern cluster” (n = 183: average posterior probability of assignment = 0.819 ± 0.156). Low posterior probabilities of assignment (0.50–0.55) were detected for only 13 individuals. Fig 3 highlights the spatial segregation of these gene pools, as well as a region of overlap near the Equator that was most obvious in a densely sampled area of the Pacific Ocean. Differentiation across this global Equatorial barrier was low but statistically significant (Table 2; AMOVA: Φ_ST_ = 0.027, *p* < 0.001).

### Historic population growth

**Fig 4:**
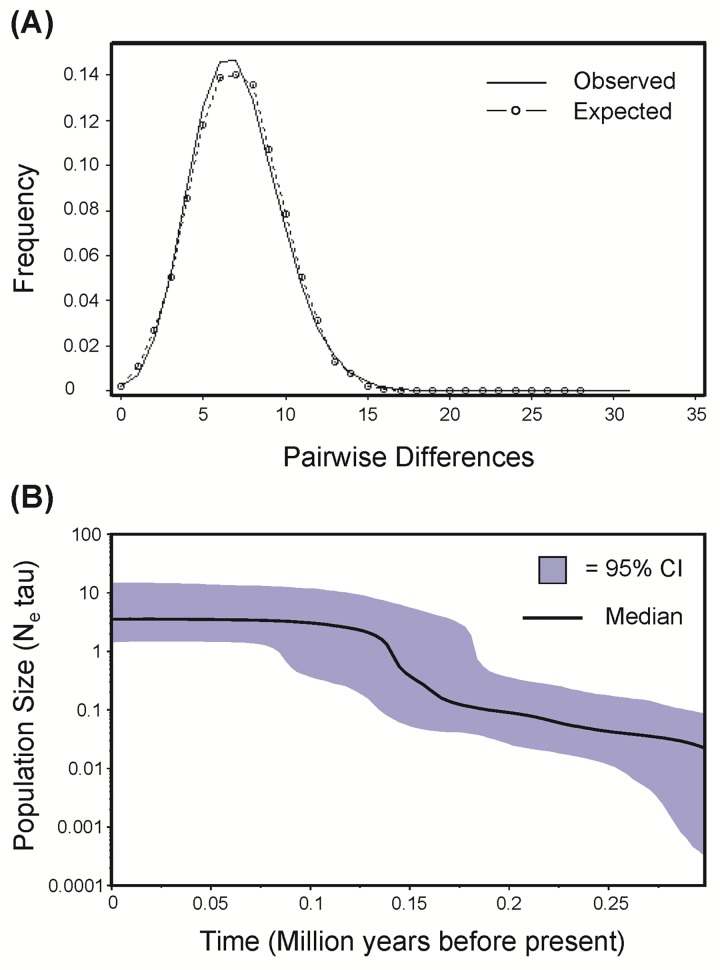
Assessments of Historical Population Growth for *Exocoetus volitans*. (**A**) Mismatch distribution of cytochrome b sequence data for all individuals (n = 266) generated using Arlequin, v.3.5 [16] and DNAsp, v.5 [12]. Solid lines indicate observed frequencies of pairwise differences and dashed lines with circles indicate expected frequencies of pairwise differences. (**B**) Bayesian skyline plot of all cytochrome b sequences generated using BEAST, v.1.6.1 [25]. The solid line indicates the median change in population size over time, and the gray area represents the 95% confidence interval (CI). Note that on the y-axis # of individuals is an estimate of population size based on N_e_τ.

Cytb mutations within *E*. *volitans* departed from null expectations for a selectively neutral gene in a single gene pool with constant *N*_*e*_. All three test statistics were statistically significant: Tajima's D (D = -2.63; p< 0.001), Fu and Li's D (D = -5.24; p<0.02) and Fu and Li's F (F = 4.64, p< 0.02; Table 3). These results are consistent with relatively recent population growth, recovery after a severe (but temporary) bottleneck, some amount of population subdivision, or natural selection. Analysis of the mismatch distribution (MMD) and Bayesian skyline plot (BSP) both supported population growth as the best interpretation. The MMD analysis resulted in a unimodal distribution that did not significantly differ from the null model of population expansion, estimated to occur 147,604 years before present (95% CI: 137,839–159,810; Fig 4A, Table 3). Similarly, the BSP analysis suggests a population expansion of approximately one order of magnitude, sometime between 125,000 and 175,000 years before present (Fig 4). Contemporary *N*_*e*_ is estimated to be on the order of 10^9^ individuals.

**Table 3:** Results of neutrality tests and mismatch distributions for each putative population of *Exocoetus volitans*.

	Tajima's D		Fu and Li's D		Fu and Li's F		Mismatch Distribution									
Putative Population	D	p-value	D	p-value	F	p-value	HRI	p-value	SSD	p-value	Θ_k_ (time = 0)	N_e_ (time = 0)	Θ_k_ (time = 1)	N_e_ (time = 1)	τ (95% CI)	t (years) (95% CI)
**Worldwide**	-2.638	<0.001	-5.248	<0.02	-4.648	<0.02	0.007	0.990	0.0030	0.020	0.029	655	63208.41	1,428,761,528	6.53 (6.098–7.070)	147,604 (137,839–159,810)
**Pacific Ocean**	-2.523	<0.001	-4.865	<0.02	-4.599	<0.02	0.006	0.659	0.0004	0.609	0.305	6,894	57139.43	1,291,578,435	7.94 (6.215–9.295)	179,476 (140,484–210,104)
**Atlantic Ocean**	-2.679	<0.001	-5.749	<0.02	-5.176	<0.02	0.009	0.939	0.0036	0.010	0.036	814	67571.62	1,527,387,432	5.58 (4.973–6.215)	126,130 (112,410–140,484)
**Indian Ocean**	-1.015	>0.10	-1.015	>0.10	-1.082	>0.10	0.190	0.516	0.0685	0.423	1.257	28,413	66096.39	1,494,041,365	6.62 (1.299–12.543)	149,638 (29,363–283,522)
**Northern cluster**	-2.671	<0.001	-5.909	<0.02	-5.197	<0.02	0.008	0.644	0.0002	0.707	0.307	6,939	49915.58	1,128,290,687	6.26 (4.518–7.701)	141,501 (102,125–174,073)
**Southern cluster**	-2.438	<0.01	-4.605	<0.02	-4.447	<0.02	0.006	0.783	0.0005	0.706	0.362	8,183	46272.12	1,045,933,996	8.01 (5.789–9.789)	181,058 (130,854–221,270)

## Discussion

Because *E*. *volitans* is short-lived and small-bodied, we hypothesized that populations separated by known biogeographic barriers would represent discrete gene pools. However, mtDNA population structure was very limited. We did detect statistically significant differentiation between the Pacific and Atlantic Oceans, as well as a global population barrier delineating gene pools north and south of the Equator.

### Circumtropical patterns of population structure in an epipelagic specialist

Despite two statistically significant barriers to global dispersal, *E*. *volitans* individuals share high genetic similarity on a global scale. Population genetic structure has been studied in two other flyingfish species with smaller distributions. Based on mtDNA data, there is no population differentiation within the Bony Flyingfish, *Hirundichthys oxycephalus*, in the northwestern Pacific (Ф_CT_ = −0.0306, p = 0.1, [29]). However, the Fourwing Flyingfish, *Hirundichthys affinis*, is subdivided into three populations or "stocks" in the tropical Atlantic (Φ_CT_ = 0.49; Φ_ST_ = 0.42 to 0.80; [30]). Life history differences between *E*. *volitans* and *Hirundichthys affinis* may explain these contrasting patterns. *E*. *volitans* has a much larger geographic range, covering > 100 million kilometers of open ocean [6], while the distribution of *H*. *affinis* is ten-fold smaller [31]. Additionally, *H*. *affinis* lives in coastal waters (neritic) particularly during early life stages [32], while *E*. *volitans* primarily occurs in epipelagic waters throughout all stages of life (holo-epipelagic) [6]. Also, *H*. *affinis* spawns in aggregations, attaching eggs to floating vegetation [29, 30] while, *E*. *volitans* broadcasts buoyant eggs [9]. Whether the reproductive behavior of *E*. *volitans* contributes to high levels of population connectivity is not fully understood because the reproductive biology of this species remains to be fully documented. Major distinctions in life history and population sizes likely confer species differences in population-level genetic variation.

Our finding of minimal global-scale population structure in *E*. *volitans* can be compared with other genetic studies involving widespread marine fishes based on mitochondrial DNA data (Table 4). A number of widespread oceanic fish species have higher Φ_ST_ values than detected in *E*. *volitans*, including swordfish (Φ_ST_ = 0.09; [33], bigeye tuna (Φ_ST_ = 0.22; [34]) and blue marlin (Φ_ST_ = 0.22; [35]). Interestingly, [8], large body size and high mobility are imperfectly correlated with Φ_ST_, since even lower Φ_ST_ values have been observed from intermediate-sized (e.g., pelagic wahoo; Φ_ST_ < 0.0001; [24]) and small-bodied species (e.g., tropical two-wing flyingfish; Φ_ST_ < 0.04; this study).

**Table 4:** Comparison of Φ_ST_ values from population genetic studies involving widely distributed marine teleosts including *Exocoetus volitans* (results of present study in bold), and two other flyingfish species (*Hirundichthys affinis*, and *H*. *oxycephalus*) for which a Φ_ST_ estimates are available.

Common Name	Scientific Name	Φ_ST_	p-value	Collection Localities	Coastal vs. Pelagic	Citation
Blacktail Snapper	*Lutjanus fulvus*	0.640	<0.001	within Indo-Pacific, including Marquesas	Coastal	[36]
Bigscale Soldierfish	*Myripristis berndti*	0.583	<0.001	Indian vs. Pacific	Coastal	[37]
Common Bluestripe Snapper	*Lutjanus kasmira*	0.300	<0.001	within Indo-Pacific, including Marquesas	Coastal	[36]
Bigeye Tuna	*Thunnus obesus*	0.220	<0.01	Atlantic vs. Indian vs. Pacific	Pelagic	[34]
Blue Marlin	*Makaira nigricans*	0.217	<0.001	Atlantic vs. Pacific	Pelagic	[35]
Swordfish	*Xiphias gladius*	0.091	<0.001	Atlantic vs. Mediterranean vs. Indo-Pacific	Pelagic	[33]
Bignose Unicornfish	*Naso vlamingii*	0.077	<0.05	Indian vs. Pacific	Coastal	[38]
Yellowfin Tuna	*Thunnus albacares*	0.070	<0.001	Atlantic vs. Pacific	Pelagic	[39]
Albacore	*Thunnus alalunga*	0.041	0.009	Mediterranean vs. Atlantic vs. Pacific	Pelagic	[40]
White Marlin	*Tetrapturus albidus*	0.040	0.045	Western North Atlantic vs. Caribbean	Pelagic	[41]
Spotted Unicornfish	*Naso brevirostris*	0.030	0.08	Indian vs. Pacific	Coastal	[42]
Bluespine Unicornfish	*Naso unicornis*	0.018	0.02	Indian vs. Pacific	Coastal	[42]
Bluefin Tuna	*Thunnus thynnus*	0.013	0.0139	West Atlantic vs. East Atlantic/ Mediterranean	Pelagic	[43]
Wahoo	*Acanthocybium solandri*	<0.0001	0.634	Global	Pelagic	[24]
Fourwing Flyingfish	*Hirundichthys affinis*	0.42–0.80	<0.001	central western Atlantic	Coastal	[30]
Bony Flyingfish	*Hirundichthys oxycephalus*	0.04	<0.05	northwest Pacific	Coastal	[29]
**Tropical Two-Wing Flyingfish**	***Exocoetus volitans***	**0.035**	**<0.001**	**Atlantic vs. Pacific**	**Pelagic**	**Present study**
**Tropical Two-Wing Flyingfish**	***Exocoetus volitans***	**0.027**	**<0.001**	**Northern cluster vs. Southern cluster**	**Pelagic**	**Present study**

Extremely large effective population sizes, that are comparable to those we estimate for *E*. *volitans*, drive nonequilibrium conditions that can result in estimates of genetic divergence that are comparable to the effects of extensive contemporary gene flow [44]. Although near-zero values of Φ_ST_ are consistent with high gene flow, they are also consistent with the persistent signature of a recent population expansion under almost any level of current gene flow [21]. Our estimates of species-wide population expansion to > 1 billion fish only ~150,000 years ago (see below) are conditions that could lead to this nonequilibrium effect on Φ_ST_ estimates. Thus, based on our data, we cannot dismiss the possibility that current gene flow across the range of *E*. *volitans* is lower than indicated by Φ_ST_ estimates [45].

### Evidence for barriers to *E*. *volitans* gene flow

Our analyses indicate at least two barriers to gene flow in *E*. *volitans*: the Isthmus of Panama barrier and a worldwide equatorial barrier (Fig 3). Small sample sizes limited our ability to adequately test whether other barriers (e.g., BB and SSB) are equally important. The effects of the Isthmus of Panama barrier and a worldwide equatorial barrier are detectable notwithstanding the potential effects of large population size and population expansion described above. The Isthmus of Panama currently represents an impermeable barrier for most marine fishes, and may have been a barrier for pelagic marine fishes for the past 15 million years [46].

The finding of comparable Φ_ST_ values (~0.03) for the Isthmian and equatorial barriers (Table 2) suggests that gene flow across the equatorial barrier is low. The worldwide equatorial barrier we propose for *E*. *volitans* has also been suggested for the pelagic copepod *Haloptilus longicornis* by Norton and Goetze [47]. These authors found a distinct genetic break at approximately 0–12°N in both the Atlantic and Pacific oceans, and hypothesized the presence of a physical or biophysical barrier restricting the latitudinal dispersal of pelagic copepods. A similar genetic subdivision has not been proposed for circumtropical fishes, although it has been discussed for a few species within ocean basins. For example, in the Atlantic, distinct swordfish populations have been detected on either side of the Equator [48]. Similarly, populations of striped marlin in the Pacific may partition in a north-to-south pattern [49]. However, hypothesizing a common cause for this divergence in multiple taxa is difficult due to variation in the biology of the species. Swordfish and marlin are large, fast-swimming, highly migratory, thermally-tolerant, and breed in specific areas, suggesting that behavior may play in role in their divergence. In contrast, for slow-swimming *E*. *volitans* and the planktonic copepod *H*. *longicornis*, oceanographic and/or biophysical barriers to dispersal are the most likely explanation for equatorial genetic divergence [47]. For *E*. *volitans*, it is possible that the species’ buoyant eggs are advected away from equatorial waters through natural processes of oceanographic upwelling and/or the Coriolis Effect.

### *E*. *volitans* historical demography

Based on the star-like mitochondrial gene genealogy (i.e., high haplotype diversity and low nucleotide diversity), the species-wide effective population size of *E*. *volitans* appears to have been smaller in the recent past. The BSP and MMD analyses both suggested dramatic population growth beginning approximately 150,000 years ago. This time frame (the middle Pleistocene) was also suggested by Chou and colleagues [29] for demographic population expansion of *H*. *oxycephalus*. These authors suggested that range and/or demographic expansion may have been driven by the end of an era of maximum cooling sea surface temperatures (SST). Indeed, sea level changes (along with SST changes) during the Pleistocene have been suggested as correlated for population bottlenecks and/or by post-glacial range expansions in many species [50–54]. The preference of *E*. *volitans* for warmer tropical waters may have shaped its demographic history, and likely continues to influence the species' contemporary patterns of gene flow. However, given our study’s focus on a single genetic marker, we cannot discount other explanations for the mitochondrial gene genealogy. An alternative interpretation is that a selective sweep occurred within this species, leading to an abundance of distal branches, creating the "fireworks"-like appearance of this genealogy (see [55]). Multilocus assessments and genetic analyses of other epipelagic specialists will allow further validation of the post-glacial range expansion hypothesis, and provide key insight regarding current patterns of epipelagic marine biodiversity.

## Supporting Information

S1 Table Specimen sampling data for *Exocoetus volitans* specimens. Museum voucher numbers (when available) and collection information are included for individuals used in genetic data analyses (n = 266). Assignments to southern or northern clusters resulting from analysis within Geneland v.1 [17] are also identified.
(PDF)
