# Supplementary material for: Population Genetic Structure of the Tropical Two-Wing Flyingfish (Exocoetus volitans)
Source: PLoS One. 2016 Oct 13;11(10):e0163198. doi: 10.1371/journal.pone.0163198 (PMC5063402; doi:10.1371/journal.pone.0163198)
Supplement: S1 Table — Museum voucher numbers (when available) and collection information are included for individuals used in genetic data analyses (n = 266). Assignments to southern or northern clusters resulting from analysis within Geneland v.1 [17] are also identified. (PDF) [file pone.0163198.s001.pdf]

Specimen #	Voucher #	Locality	Latitude (Dec Deg)	Longitude (Dec Deg)	Date Collected (YYYY MM DD)	Population	Prob Pop A	Prob Pop B	Genbank # (cytb)
1585	SIO-07-132	Pacific	10.18° N	104.53° W	N/A	B	0.04	0.96	HQ325634
1586	SIO-07-132	Pacific	10.18° N	104.53° W	N/A	B	0.04	0.96	HQ325635
1856	USNM-380577	Atlantic	4.68° N	22.33° W	2002 09 (01-02)	A	0.57	0.43	pending
1858	USNM-380582	Atlantic	8.26° S	20.98° W	2002 09 06	A	0.56	0.44	pending
1861	USNM-380581	Atlantic	8.19° S	21.92° W	2002 09 17	A	0.57	0.43	pending
1862	USNM-380581	Atlantic	8.19° S	21.92° W	2002 09 17	A	0.57	0.43	pending
1863	USNM-380581	Atlantic	8.19° S	21.92° W	2002 09 17	A	0.57	0.43	pending
3708	ROM #79239	Pacific	2.68° S	91.12° W	2006 09 25	A	0.59	0.41	pending
3709	ROM #79239	Pacific	2.68° S	91.12° W	2006 09 25	A	0.59	0.41	pending
3805	ROM #79315	Pacific	5.38° N	99.53° W	2006 10 23	A	0.81	0.19	pending
3828	ROM #79227	Pacific	1.17° S	082.45° W	2006 09 28	A	0.59	0.41	pending
3829	ROM #79227	Pacific	1.17° S	082.45° W	2006 09 28	A	0.59	0.41	pending
3833	ROM #79261	Pacific	1.17° S	082.45° W	2006 09 28	A	0.59	0.41	pending
3864	pending	Pacific	5.32° N	137.63° W	2006 09 09	B	0.15	0.85	pending
3865	pending	Pacific	5.32° N	137.63° W	2006 09 09	B	0.15	0.85	pending
3871	ROM #79202	Pacific	2.20° S	96.28° W	2006 10 16	A	0.59	0.41	pending
3872	ROM #79202	Pacific	2.20° S	96.28° W	2006 10 16	A	0.59	0.41	pending
4131	pending	Pacific	6.15° N	92.20° W	2006 08 30	A	0.77	0.23	pending
4183	ROM #79300	Pacific	5.65° N	108.00° W	2006 08 25	B	0.13	0.87	pending
4206	pending	Pacific	7.25° N	101.37° W	2006 08 27	B	0.03	0.97	pending
4210	pending	Pacific	7.25° N	101.37° W	2006 08 27	B	0.03	0.97	pending
4269	ROM #79200	Pacific	14.98° N	112.75° W	2006 11 29	B	0.08	0.92	pending
4270	ROM #79200	Pacific	14.98° N	112.75° W	2006 11 29	B	0.08	0.92	pending
4274	ROM #79198	Pacific	17.88° N	115.77° W	2006 12 01	B	0.13	0.87	pending
4275	ROM #79198	Pacific	17.88° N	115.77° W	2006 12 01	B	0.13	0.87	pending
4288	ROM #79201	Pacific	6.48° N	104.38° W	2006 08 26	A	0.69	0.31	pending
4289	ROM #79201	Pacific	6.48° N	104.38° W	2006 08 26	A	0.69	0.31	pending
4313	ROM #79218	Pacific	16.98° N	113.40° W	2006 11 30	B	0.18	0.82	pending
4572	pending	Pacific	7.63° N	97.92° W	2006 10 22	B	0.46	0.54	pending
4595	ROM #79281	Pacific	2.78° S	96.37° W	2006 09 23	A	0.59	0.41	pending
4910	ROM #79292	Pacific	8.37° N	89.47° W	2006 09 21	B	0.05	0.95	pending
5041	pending	Pacific	6.33° N	101.73° W	2006 10 13	B	0.12	0.88	pending
5042	pending	Pacific	6.33° N	101.73° W	2006 10 13	B	0.12	0.88	pending
5062	ROM #79310	Pacific	6.97° N	103.17° W	2006 10 14	A	0.62	0.38	pending
5063	ROM #79310	Pacific	6.97° N	103.17° W	2006 10 14	A	0.62	0.38	pending
5141	ROM #79313	Pacific	8.18° N	100.18° W	2006 10 12	B	0.12	0.88	pending
5142	pending	Pacific	8.18° N	100.18° W	2006 10 12	B	0.12	0.88	pending
5185	ROM #79271	Pacific	9.02° N	93.05° W	2006 10 05	B	0.14	0.86	pending
5195	ROM #79219	Pacific	8.83° N	94.42° W	2006 10 06	B	0.11	0.89	pending
5196	ROM #79219	Pacific	8.83° N	94.42° W	2006 10 06	B	0.11	0.89	pending
5235	pending	Pacific	8.25° N	113.17° W	2006 08 23	B	0.04	0.96	pending
5236	pending	Pacific	8.25° N	113.17° W	2006 08 23	B	0.04	0.96	pending
5247	ROM #79294	Pacific	6.07° N	110.70° W	2006 08 24	A	0.78	0.22	pending
5248	ROM #79294	Pacific	6.07° N	110.70° W	2006 08 24	A	0.78	0.22	pending
5280	ROM #79295	Pacific	10.35° N	115.60° W	2006 08 22	B	0.03	0.97	pending
5281	ROM #79295	Pacific	10.35° N	115.60° W	2006 08 22	B	0.03	0.97	pending
5590	pending	Pacific	2.40° S	100.93° W	2006 09 22	A	0.59	0.41	pending
5600	ROM #79232	Pacific	4.50° N	134.75° W	2006 09 10	A	0.80	0.20	pending
5601	pending	Pacific	4.50° N	134.75° W	2006 09 10	A	0.80	0.20	pending
5608	ROM #79272	Pacific	7.35° S	98.43° W	2006 10 14	A	0.56	0.44	pending
5629	ROM #79224	Pacific	14.87° N	159.67° W	2005 09 06	B	0.05	0.95	pending
5647	ROM #79230	Pacific	3.85° N	163.18° W	2005 08 22	A	0.56	0.44	pending
5682	ROM #79266	Pacific	12.23° N	168.53° W	2005 10 06	B	0.09	0.91	pending
5685	ROM #79293	Pacific	8.68° N	164.82° W	2005 08 15	B	0.19	0.81	pending
5686	ROM #79293	Pacific	8.68° N	164.82° W	2005 08 15	B	0.19	0.81	pending
5692	ROM #79301	Pacific	8.03° N	162.38° W	2005 08 20	B	0.47	0.53	pending
5693	ROM #79220	Pacific	7.88° N	161.32° W	2005 08 27	B	0.01	0.99	pending
5694	ROM #79220	Pacific	7.88° N	161.32° W	2005 08 27	B	0.01	0.99	pending
5695	ROM #79214	Pacific	11.93° N	170.08° W	2005 10 05	B	0.08	0.92	pending
5696	ROM #79214	Pacific	11.93° N	170.08° W	2005 10 05	B	0.08	0.92	pending
5702	ROM #79302	Pacific	5.77° N	164.18° W	2005 08 29	A	0.82	0.18	pending
5703	ROM #79302	Pacific	5.77° N	164.18° W	2005 08 29	A	0.82	0.18	pending
5704	ROM #79215	Pacific	13.42° N	160.35° W	2005 09 05	B	0.03	0.97	pending
5705	ROM #79303	Pacific	4.27° N	163.57° W	2005 08 21	A	0.60	0.40	pending
5706	ROM #79222	Pacific	4.95° N	161.93° W	2005 08 23	B	0.15	0.85	pending
5707	ROM #79222	Pacific	4.95° N	161.93° W	2005 08 23	B	0.15	0.85	pending
5720	ROM #79284	Pacific	20.60° N	157.95° W	2005 09 08	B	0.40	0.60	pending
5721	ROM #79284	Pacific	20.60° N	157.95° W	2005 09 08	B	0.40	0.60	pending
5728	ROM #79325	Pacific	14.22° N	169.70° W	2005 10 04	B	0.10	0.90	pending
5733	ROM #79308	Pacific	8.92° N	164.03° W	2005 09 02	B	0.21	0.79	pending
5743	pending	Pacific	1.42° S	113.70° W	2006 09 17	A	0.59	0.41	pending
5773	pending	Pacific	6.22° N	117.85° W	2006 08 09	B	0.44	0.56	pending
5782	ROM #79226	Pacific	5.02° N	113.58° W	2006 10 28	B	0.13	0.87	pending
5783	pending	Pacific	5.02° N	113.58° W	2006 10 28	B	0.13	0.87	pending
5800	ROM #79225	Pacific	16.73° N	118.50° W	2006 12 01	B	0.12	0.88	pending
5810	ROM #79235	Pacific	3.37° N	110.85° W	2006 10 27	B	0.41	0.59	pending
5811	ROM #79235	Pacific	3.37° N	110.85° W	2006 10 27	B	0.41	0.59	pending
5842	ROM #79309	Pacific	8.55° N	108.78° W	2006 11 24	B	0.37	0.63	pending
5862	ROM #79316	Pacific	17.10° N	150.37° W	2006 08 23	B	0.05	0.95	pending
5863	pending	Pacific	17.10° N	150.37° W	2006 08 23	B	0.05	0.95	pending
5937	ROM #79255	Pacific	9.98° N	164.98° W	2005 10 30	B	0.03	0.97	pending
5938	ROM #79255	Pacific	9.98° N	164.98° W	2005 10 30	B	0.03	0.97	pending
5950	ROM #79229	Pacific	14.12° N	164.78° W	2005 10 24	B	0.08	0.92	pending
5951	ROM #79229	Pacific	14.12° N	164.78° W	2005 10 24	B	0.08	0.92	pending

Specimen #	Voucher #	Locality	Latitude (Dec Deg)	Longitude (Dec Deg)	Date Collected (YYYY MM DD)	Population	Prob Pop A	Prob Pop B	Genbank # (cytb)
5958	ROM #79269	Pacific	11.68° N	162.73° W	2005 10 31	B	0.07	0.93	pending
5967	ROM #79282	Pacific	16.07° N	163.22° W	2005 10 26	B	0.07	0.93	pending
5968	ROM #79282	Pacific	16.07° N	163.22° W	2005 10 26	B	0.07	0.93	pending
5980	ROM #79321	Pacific	16.05° N	163.43° W	2005 10 25	B	0.07	0.93	pending
5981	ROM #79321	Pacific	16.05° N	163.43° W	2005 10 25	B	0.07	0.93	pending
5983	ROM #79323	Pacific	8.87° N	160.97° W	2005 11 05	B	0.10	0.90	pending
5984	ROM #79323	Pacific	8.87° N	160.97° W	2005 11 05	B	0.10	0.90	pending
5985	ROM #79304	Pacific	5.87° N	162.20° W	2005 11 05	A	0.69	0.31	pending
5986	ROM #79304	Pacific	5.87° N	162.20° W	2005 11 05	A	0.69	0.31	pending
6109	ROM #79306	Pacific	11.08° N	144.57° E	2007 01 22	B	0.14	0.86	pending
6130	pending	Pacific	15.10° N	141.95° E	2007 02 23	B	0.09	0.91	pending
6225	ROM #88449	Pacific	14.83° N	107.10° W	2007 09 19	B	0.09	0.91	pending
6298	ROM #79288	Pacific	13.73° N	103.65° W	2007 10 20	B	0.10	0.90	pending
6299	ROM #79288	Pacific	13.73° N	103.65° W	2007 10 20	B	0.10	0.90	pending
6300	ROM #79288	Pacific	13.73° N	103.65° W	2007 10 20	B	0.10	0.90	pending
6320	ROM #79263	Pacific	16.10° N	106.25° W	2007 10 04	B	0.04	0.96	pending
6324	ROM #79263	Pacific	16.10° N	106.25° W	2007 10 04	B	0.04	0.96	pending
6352	ROM #79327	Pacific	12.47° N	104.50° W	2007 09 27	B	0.09	0.91	pending
6408	ROM #79286	Pacific	13.73° N	105.35° W	20 07 10 02	B	0.05	0.95	pending
6535	ROM #79311	Pacific	15.07° N	106.87° W	2007 08 27	B	0.08	0.92	pending
6536	ROM #79311	Pacific	15.07° N	106.87° W	2007 08 27	B	0.08	0.92	pending
6637	pending	Pacific	15.00° N	105.50° W	2007 09 25	B	0.06	0.94	pending
6821	ROM #79262	Pacific	12.47° N	104.48° W	2007 11 11	B	0.09	0.91	pending
6929	ROM #79267	Pacific	6.13° N	145.92° W	1998 09 06	A	0.76	0.24	pending
6930	ROM #79267	Pacific	6.13° N	145.92° W	1998 09 06	A	0.76	0.24	pending
7432	pending	Atlantic	0.92° N	25.41° W	2010 08 03	A	0.62	0.38	pending
7433	pending	Atlantic	0.92° N	25.41° W	2010 08 03	A	0.62	0.38	pending
7434	pending	Atlantic	0.64° N	23.98° W	2010 08 03	A	0.85	0.15	pending
7435	pending	Atlantic	5.50° N	21.28° W	2010 08 06	A	0.85	0.15	pending
7445	pending	Atlantic	5.55° N	21.27° W	2010 08 06	A	0.84	0.16	pending
7448	pending	Atlantic	0.48° N	23.11° W	2010 08 03	A	0.92	0.08	pending
7449	pending	Atlantic	0.48° N	23.11° W	2010 08 03	A	0.92	0.08	pending
7450	pending	Atlantic	0.48° N	23.11° W	2010 08 03	A	0.92	0.08	pending
7454	pending	Atlantic	5.67° N	18.84° W	2010 08 15	A	0.72	0.28	pending
7459	pending	Atlantic	5.00° N	21.24° W	2010 08 09	A	0.96	0.04	pending
7460	pending	Atlantic	5.00° N	21.24° W	2010 08 09	A	0.96	0.04	pending
7463	pending	Atlantic	0.45° N	23.05° W	2010 08 04	A	0.92	0.08	pending
7464	pending	Atlantic	0.45° N	23.05° W	2010 08 04	A	0.92	0.08	pending
7465	pending	Atlantic	0.45° N	23.05° W	2010 08 04	A	0.92	0.08	pending
7466	pending	Atlantic	0.45° N	23.05° W	2010 08 04	A	0.92	0.08	pending
7467	pending	Atlantic	0.45° N	23.05° W	2010 08 04	A	0.92	0.08	pending
7468	pending	Atlantic	0.45° N	23.05° W	2010 08 04	A	0.92	0.08	pending
7469	pending	Atlantic	0.45° N	23.05° W	2010 08 04	A	0.92	0.08	pending
7470	pending	Atlantic	0.45° N	23.05° W	2010 08 04	A	0.92	0.08	pending
7471	pending	Atlantic	0.45° N	23.05° W	2010 08 04	A	0.92	0.08	pending
7472	pending	Atlantic	0.45° N	23.05° W	2010 08 04	A	0.92	0.08	pending
7473	pending	Atlantic	0.45° N	23.05° W	2010 08 04	A	0.92	0.08	pending
7474	pending	Atlantic	0.45° N	23.05° W	2010 08 04	A	0.92	0.08	pending
7475	pending	Atlantic	0.45° N	23.05° W	2010 08 04	A	0.92	0.08	pending
7476	pending	Atlantic	0.45° N	23.05° W	2010 08 04	A	0.92	0.08	pending
7477	pending	Atlantic	0.45° N	23.05° W	2010 08 04	A	0.92	0.08	pending
7515	pending	Atlantic	4.58° N	24.48° W	2010 08 12	A	0.62	0.38	pending
7516	pending	Atlantic	4.58° N	24.48° W	2010 08 12	A	0.62	0.38	pending
7517	pending	Atlantic	4.58° N	24.48° W	2010 08 12	A	0.62	0.38	pending
7518	pending	Atlantic	4.58° N	24.48° W	2010 08 12	A	0.62	0.38	pending
7519	pending	Atlantic	4.58° N	24.48° W	2010 08 12	A	0.62	0.38	pending
7520	pending	Atlantic	4.58° N	24.48° W	2010 08 12	A	0.62	0.38	pending
7522	pending	Atlantic	3.63° N	21.76° W	2010 08 06	A	0.60	0.40	pending
7523	pending	Atlantic	3.63° N	21.76° W	2010 08 06	A	0.60	0.40	pending
7525	pending	Atlantic	5.79° N	21.16° W	2010 08 06	A	0.81	0.19	pending
7526	pending	Atlantic	5.79° N	21.16° W	2010 08 06	A	0.81	0.19	pending
7527	pending	Atlantic	5.79° N	21.16° W	2010 08 06	A	0.81	0.19	pending
7530	pending	Atlantic	5.79° N	21.16° W	2010 08 06	A	0.81	0.19	pending
7531	pending	Atlantic	5.79° N	21.16° W	2010 08 06	A	0.81	0.19	pending
7532	pending	Atlantic	5.79° N	21.16° W	2010 08 06	A	0.81	0.19	pending
7533	pending	Atlantic	5.79° N	21.16° W	2010 08 06	A	0.81	0.19	pending
7534	pending	Atlantic	5.79° N	21.16° W	2010 08 06	A	0.81	0.19	pending
7535	pending	Atlantic	5.79° N	21.16° W	2010 08 06	A	0.81	0.19	pending
7539	pending	Atlantic	5.79° N	21.16° W	2010 08 06	A	0.81	0.19	pending
7541	pending	Atlantic	5.79° N	21.16° W	2010 08 06	A	0.81	0.19	pending
7563	pending	Atlantic	4.58° N	24.48° W	2010 08 11	A	0.62	0.38	pending
7564	pending	Atlantic	4.58° N	24.48° W	2010 08 11	A	0.62	0.38	pending
7565	pending	Atlantic	4.58° N	24.48° W	2010 08 11	A	0.62	0.38	pending
7566	pending	Atlantic	4.58° N	24.48° W	2010 08 11	A	0.62	0.38	pending
7572	pending	Atlantic	6.89° N	20.92° W	2010 08 07	A	0.51	0.49	pending
7579	pending	Atlantic	5.00° N	21.24° W	2010 08 09	A	0.96	0.04	pending
7581	pending	Atlantic	5.00° N	21.24° W	2010 08 09	A	0.96	0.04	pending
7583	pending	Atlantic	5.00° N	21.24° W	2010 08 09	A	0.96	0.04	pending
7584	pending	Atlantic	5.00° N	21.24° W	2010 08 09	A	0.96	0.04	pending
7587	pending	Atlantic	5.00° N	21.24° W	2010 08 09	A	0.96	0.04	pending
7593	pending	Atlantic	5.00° N	21.24° W	2010 08 09	A	0.96	0.04	pending
7595	pending	Atlantic	5.00° N	21.24° W	2010 08 09	A	0.96	0.04	pending
7597	pending	Atlantic	5.00° N	21.24° W	2010 08 09	A	0.96	0.04	pending
7599	pending	Atlantic	5.00° N	21.24° W	2010 08 09	A	0.96	0.04	pending

Specimen #	Voucher #	Locality	Latitude (Dec Deg)	Longitude (Dec Deg)	Date Collected (YYYY MM DD)	Population	Prob Pop A	Prob Pop B	Genbank # (cytb)
7725	pending	Atlantic	5.00° N	21.24° W	2010 08 09	A	0.96	0.04	pending
7727	pending	Atlantic	5.00° N	21.24° W	2010 08 09	A	0.96	0.04	pending
7728	pending	Atlantic	5.00° N	21.24° W	2010 08 09	A	0.96	0.04	pending
7741	pending	Atlantic	5.00° N	21.24° W	2010 08 09	A	0.96	0.04	pending
7744	pending	Atlantic	5.00° N	21.24° W	2010 08 09	A	0.96	0.04	pending
7746	pending	Atlantic	5.00° N	21.24° W	2010 08 09	A	0.96	0.04	pending
7747	pending	Atlantic	5.00° N	21.24° W	2010 08 09	A	0.96	0.04	pending
7748	pending	Atlantic	5.00° N	21.24° W	2010 08 09	A	0.96	0.04	pending
7749	pending	Atlantic	5.00° N	21.24° W	2010 08 09	A	0.96	0.04	pending
7750	pending	Atlantic	5.00° N	21.24° W	2010 08 09	A	0.96	0.04	pending
7751	pending	Atlantic	5.00° N	21.24° W	2010 08 09	A	0.96	0.04	pending
7752	pending	Atlantic	5.00° N	21.24° W	2010 08 09	A	0.96	0.04	pending
7753	pending	Atlantic	5.00° N	21.24° W	2010 08 09	A	0.96	0.04	pending
7781	pending	Atlantic	4.94° N	21.24° W	2010 08 (08 - 09)	A	0.96	0.04	pending
7783	pending	Atlantic	4.94° N	21.24° W	2010 08 (08 - 09)	A	0.96	0.04	pending
7784	pending	Atlantic	4.94° N	21.24° W	2010 08 (08 - 09)	A	0.96	0.04	pending
7785	pending	Atlantic	4.94° N	21.24° W	2010 08 (08 - 09)	A	0.96	0.04	pending
7792	pending	Atlantic	4.94° N	21.24° W	2010 08 (08 - 09)	A	0.96	0.04	pending
7793	pending	Atlantic	4.94° N	21.24° W	2010 08 (08 - 09)	A	0.96	0.04	pending
7794	pending	Atlantic	4.94° N	21.24° W	2010 08 (08 - 09)	A	0.96	0.04	pending
7796	pending	Atlantic	4.94° N	21.24° W	2010 08 (08 - 09)	A	0.96	0.04	pending
7797	pending	Atlantic	4.94° N	21.24° W	2010 08 (08 - 09)	A	0.96	0.04	pending
7799	pending	Atlantic	4.94° N	21.24° W	2010 08 (08 - 09)	A	0.96	0.04	pending
7800	pending	Atlantic	4.94° N	21.24° W	2010 08 (08 - 09)	A	0.96	0.04	pending
7810	pending	Atlantic	4.94° N	21.24° W	2010 08 (08 - 09)	A	0.96	0.04	pending
7811	pending	Atlantic	4.94° N	21.24° W	2010 08 (08 - 09)	A	0.96	0.04	pending
7812	pending	Atlantic	4.94° N	21.24° W	2010 08 (08 - 09)	A	0.96	0.04	pending
7813	pending	Atlantic	4.94° N	21.24° W	2010 08 (08 - 09)	A	0.96	0.04	pending
7821	pending	Atlantic	4.94° N	21.24° W	2010 08 (08 - 09)	A	0.96	0.04	pending
7825	pending	Atlantic	4.94° N	21.24° W	2010 08 (08 - 09)	A	0.96	0.04	pending
7826	pending	Atlantic	4.94° N	21.24° W	2010 08 (08 - 09)	A	0.96	0.04	pending
7827	pending	Atlantic	4.94° N	21.24° W	2010 08 (08 - 09)	A	0.96	0.04	pending
7828	pending	Atlantic	4.94° N	21.24° W	2010 08 (08 - 09)	A	0.96	0.04	pending
7830	pending	Atlantic	4.94° N	21.24° W	2010 08 (08 - 09)	A	0.96	0.04	pending
7842	pending	Atlantic	4.94° N	21.24° W	2010 08 (08 - 09)	A	0.96	0.04	pending
7843	pending	Atlantic	4.94° N	21.24° W	2010 08 (08 - 09)	A	0.96	0.04	pending
7844	pending	Atlantic	4.94° N	21.24° W	2010 08 (08 - 09)	A	0.96	0.04	pending
7845	pending	Atlantic	4.94° N	21.24° W	2010 08 (08 - 09)	A	0.96	0.04	pending
7848	pending	Atlantic	0.46° N	23.08° W	2010 08 (03 - 04)	A	0.92	0.08	pending
7858	pending	Atlantic	0.46° N	23.08° W	2010 08 (03 - 04)	A	0.92	0.08	pending
7878	pending	Atlantic	0.46° N	23.08° W	2010 08 (03 - 04)	A	0.92	0.08	pending
7883	pending	Atlantic	0.46° N	23.08° W	2010 08 (03 - 04)	A	0.92	0.08	pending
7885	pending	Atlantic	0.46° N	23.08° W	2010 08 (03 - 04)	A	0.92	0.08	pending
7886	pending	Atlantic	0.46° N	23.08° W	2010 08 (03 - 04)	A	0.92	0.08	pending
7891	pending	Atlantic	0.46° N	23.08° W	2010 08 (03 - 04)	A	0.92	0.08	pending
7892	pending	Atlantic	0.46° N	23.08° W	2010 08 (03 - 04)	A	0.92	0.08	pending
7894	pending	Atlantic	0.46° N	23.08° W	2010 08 (03 - 04)	A	0.92	0.08	pending
7895	pending	Atlantic	0.46° N	23.08° W	2010 08 (03 - 04)	A	0.92	0.08	pending
7897	pending	Atlantic	0.46° N	23.08° W	2010 08 (03 - 04)	A	0.92	0.08	pending
7898	pending	Atlantic	0.46° N	23.08° W	2010 08 (03 - 04)	A	0.92	0.08	pending
7900	pending	Atlantic	0.46° N	23.08° W	2010 08 (03 - 04)	A	0.92	0.08	pending
7902	pending	Atlantic	0.46° N	23.08° W	2010 08 (03 - 04)	A	0.92	0.08	pending
7903	pending	Atlantic	0.46° N	23.08° W	2010 08 (03 - 04)	A	0.92	0.08	pending
7905	pending	Atlantic	0.46° N	23.08° W	2010 08 (03 - 04)	A	0.92	0.08	pending
7907	pending	Atlantic	0.46° N	23.08° W	2010 08 (03 - 04)	A	0.92	0.08	pending
7911	pending	Atlantic	0.46° N	23.08° W	2010 08 (03 - 04)	A	0.92	0.08	pending
7912	pending	Atlantic	0.46° N	23.08° W	2010 08 (03 - 04)	A	0.92	0.08	pending
7913	pending	Atlantic	0.46° N	23.08° W	2010 08 (03 - 04)	A	0.92	0.08	pending
7914	pending	Atlantic	0.46° N	23.08° W	2010 08 (03 - 04)	A	0.92	0.08	pending
7928	pending	Atlantic	0.46° N	23.08° W	2010 08 (03 - 04)	A	0.92	0.08	pending
7932	pending	Atlantic	4.58° N	24.48° W	2010 08 (11 - 12)	A	0.62	0.38	pending
7933	pending	Atlantic	4.58° N	24.48° W	2010 08 (11 - 12)	A	0.62	0.38	pending
7935	pending	Atlantic	4.58° N	24.48° W	2010 08 (11 - 12)	A	0.62	0.38	pending
7936	pending	Atlantic	4.58° N	24.48° W	2010 08 (11 - 12)	A	0.62	0.38	pending
7964	pending	Atlantic	4.58° N	24.48° W	2010 08 (11 - 12)	A	0.62	0.38	pending
8303	pending	Atlantic	4.58° N	24.48° W	2010 08 (11 - 12)	A	0.62	0.38	pending
8357	pending	Atlantic	4.58° N	24.48° W	2010 08 (11 - 12)	A	0.62	0.38	pending
8385	pending	Atlantic	0.48° N	23.06° W	2010 08 04	A	0.92	0.08	pending
8386	pending	Atlantic	0.48° N	23.06° W	2010 08 04	A	0.92	0.08	pending
8387	pending	Atlantic	0.48° N	23.06° W	2010 08 04	A	0.92	0.08	pending
8388	pending	Atlantic	0.48° N	23.06° W	2010 08 04	A	0.92	0.08	pending
8389	pending	Atlantic	0.48° N	23.06° W	2010 08 04	A	0.92	0.08	pending
8390	pending	Atlantic	0.48° N	23.06° W	2010 08 04	A	0.92	0.08	pending
8391	pending	Atlantic	0.48° N	23.06° W	2010 08 04	A	0.92	0.08	pending
8393	pending	Atlantic	0.48° N	23.06° W	2010 08 04	A	0.92	0.08	pending
8395	pending	Atlantic	0.48° N	23.06° W	2010 08 04	A	0.92	0.08	pending
8397	pending	Atlantic	0.48° N	23.06° W	2010 08 04	A	0.92	0.08	pending
8399	pending	Atlantic	0.48° N	23.06° W	2010 08 04	A	0.92	0.08	pending
8401	pending	Atlantic	0.48° N	23.06° W	2010 08 04	A	0.92	0.08	pending
8402	pending	Atlantic	0.48° N	23.06° W	2010 08 04	A	0.92	0.08	pending
8403	pending	Atlantic	0.48° N	23.06° W	2010 08 04	A	0.92	0.08	pending
8408	pending	Atlantic	0.48° N	23.06° W	2010 08 04	A	0.92	0.08	pending
8439	pending	Pacific	21.42° N	158.18° W	2010 09 06	B	0.44	0.56	pending
8440	pending	Pacific	21.42° N	158.18° W	2010 09 06	B	0.44	0.56	pending

Specimen #	Voucher #	Locality	Latitude (Dec Deg)	Longitude (Dec Deg)	Date Collected (YYYY MM DD)	Population	Prob Pop A	Prob Pop B	Genbank # (cytb)
8454	pending	Pacific	25.07° N	164.35° W	2010 09 09	A	0.51	0.49	pending
8455	pending	Pacific	25.07° N	164.35° W	2010 09 09	A	0.51	0.49	pending
8456	pending	Pacific	25.07° N	164.35° W	2010 09 09	A	0.51	0.49	pending
8478	pending	Pacific	23.62° N	161.67° W	2010 09 08	B	0.47	0.53	pending
8519	pending	Pacific	13.98° N	145.87° E	2007 02 20	B	0.04	0.96	pending
922054	pending	Atlantic	29.00° N	86.50° W	1992 04 26	A	0.51	0.49	pending
951240	pending	Indian	22.23° N	60.73° E	1995 05 21	B	0.46	0.54	pending
951270	pending	Indian	3.13° S	55.00° E	1995 06 30	A	0.58	0.42	pending
951296	pending	Indian	13.00° N	57.08° E	1995 05 11	B	0.08	0.92	pending
951554	pending	Indian	5.00° N	49.03° E	1995 05 03	B	0.30	0.70	pending
960051	pending	Atlantic	26.00° N	91.50° W	1996 05 21	A	0.51	0.49	pending
960129	pending	Atlantic	27.78° N	84.47° W	1996 05 30	A	0.51	0.49	pending
970048	pending	Indian	26.13° S	86.60° E	1997 03 13	A	0.52	0.48	pending
020663	pending	Atlantic	32.98° N	76.33° W	2002 03 25	B	0.50	0.50	pending
